# FGF21 analogue PF-05231023 on alcohol consumption and neuronal activity in the nucleus accumbens

**DOI:** 10.1038/s41386-025-02133-z

**Published:** 2025-05-26

**Authors:** Bart J. Cooley, Cassandra V. Occelli Hanbury-Brown, Eun A. Choi, Willow A. Heller, Alyssa W. Lim, Andrew J. Lawrence, Paul S. Haber, Gavan P. McNally, E. Zayra Millan

**Affiliations:** 1 School of Psychology, UNSW Sydney, Sydney, NSW, Australia; 2 Florey Institute of Neuroscience and Mental Health, Parkville, Australia; Florey Department of Neuroscience and Mental Health, University of Melbourne, Melbourne, VIC, Australia; 3 Faculty of Medicine and Health, Sydney Medical School, The University of Sydney, Sydney, NSW Australia; 4 Edith Collins Centre, Drug Health Services, Royal Prince Alfred Hospital, Camperdown, NSW Australia

**Keywords:** Addiction, Reward, Motivation

## Abstract

Fibroblast growth factor 21 (FGF21) is a liver-derived hormone known to suppress alcohol consumption in mice and non-human primates. However, the role of FGF21 in modulating environmental and behavioural factors driving alcohol consumption—such as cue-driven responses and effortful actions to obtain alcohol—and its effects on neural activity related to consumption, remain unclear. Here, we evaluated the impact of PF-05231023, a long-acting FGF21 analogue, across multiple dimensions of alcohol consumption and motivation and examined consumption-related activity in the nucleus accumbens. PF-05231023 reduced alcohol intake and preference in a dose- and sex-specific manner; diminished approach behaviours following an alcohol but not sucrose cue; and decreased lever-pressing under a progressive-ratio schedule, both alone and when combined with the Glucagon-like peptide-1 (GLP-1) agonist Exendin-4; it did not reduce lever-pressing for sucrose in alcohol-naïve mice. Additionally, PF-05231023 altered the microstructure of alcohol consumption by shortening drinking bouts and increased the recruitment of nucleus accumbens (Acb) neurons associated with bout termination as determined by micro-endoscopy of GCaMP7f. These findings demonstrate that PF-05231023 broadly suppresses alcohol-motivated behaviours without impacting natural reward and that targeting FGF21 signaling in combination with GLP-1 agonists may enhance therapeutic efficacy. Mechanistically, the observed reductions in alcohol consumption following PF-05231023 may involve diminished alcohol palatability and modulation of neuronal activity from distinct subsets of Acb neurons.

## Introduction

Alcohol use and related harms are a leading cause of global morbidity [1]. Medications for alcohol use disorder (AUD) like naltrexone, nalmefene, acamprosate, and topiramate are available, but their effectiveness varies widely, leaving a significant gap in treatment options [2, 3]. Emerging pharmacological targets include peptides that promote metabolic health (glucose homeostasis, insulin sensitivity) and energy balance, such as those secreted by the gut (e.g., GLP-1, ghrelin [4–7]), brain (e.g., orexin [8–10]), and adipose tissue (e.g., leptin [11]). These peptides exploit shared mechanisms between the regulation of alcohol-motivated behaviour and satiety pathways [11, 12], but there is emerging interest in fibroblast growth factor 21 (FGF21)—a liver-secreted endocrine peptide that regulates macronutrient preference [13–15]—as a promising new target, complementary to the actions of other energy-balancing peptides [16, 17], for reducing alcohol consumption.

FGF21 is induced in the liver in response to various metabolic stressors, including alcohol [18–20]. It signals via binding to an FGFR1c/β-klotho (KLB) cell surface receptor complex [21, 22]. While it acts at multiple peripheral tissues, it can also act centrally to bias macronutrient choice [15], indicating its potential to directly regulate appetitive behaviour, though the extent of this regulation for conditions involving alcohol consumption are not well characterised. Three lines of evidence support the role for FGF21 in the regulation of alcohol consumption. First, overexpression of FGF21 in transgenic mouse lines significantly reduces alcohol preference [23] while pharmacological administration of FGF21 or its analogue, PF-05231023, reduces alcohol consumption in mice and alcohol-preferring vervet monkeys [24]. Second, in large-scale genome wide association studies in humans, single nucleotide polymorphisms (SNPs) in *FGF21* and *KLB* regulatory genes were significantly associated with alcohol consumption and AUD risk [25–27]. Finally, disruption of central FGF21 signalling via brain-specific knock out of the obligate FGF21 coreceptor, KLB, increased alcohol consumption in mice [27].

The central actions of FGF21 on alcohol consumption have been localised to an amygdala-ventral striatal pathway involving KLB+ neurons in the basolateral amygdala (BLA) that project to the nucleus accumbens (Acb [24]), consistent with prior studies implicating this pathway in regulating alcohol intake and cue-driven conditioned responses in the presence of alcohol-paired cues [28]. The Acb in particular is centrally positioned to rapidly suppress intake of orally-ingested rewards, with previous studies showing that stimulation of the Acb or its projections inhibits consumption [29, 30]. Conversely, pauses in the activity of Acb cells are required to initiate and maintain consumption [29, 30], supporting its role as a gateway for consummatory behaviour. Whether and how FGF21 acts directly on individual Acb cells to diminish these pauses remains unknown.

The evidence implicating FGF21 or its analogues over the regulation of alcohol consumption is important but is broadly limited to consumption per se. The influence of FGF21 on precursors to drinking, including approach triggered by alcohol-associated cues and instrumental actions required to obtain alcohol remain unknown. We addressed these here and first show that PF-05231023 reduces voluntary alcohol consumption and preference in male but not female mice in a dose-dependent manner. PF-05231023 also attenuates responses following the presentation of an alcohol- but not sucrose-predictive cue and reduces the motivation to seek alcohol on an instrumental progressive ratio (PR) test. To understand how PF-05231023 suppresses alcohol consumption we examined the microstructure of licking in alcohol drinking mice while recording the activity of individual neurons in the Acb. We found that PF-05231023 altered licking microstructure possibly indicating reduced alcohol palatability and modulated specific subsets of Acb cells associated with initiating and terminating consumption.

## Materials and methods

### Animals

All procedures were approved by the Animal Care and Ethics Committee at UNSW Sydney and conformed to ARRIVE guidelines. C57BL/6 J mice (OzGene, Perth, Australia) were single- housed on a 12:12-hour light/dark cycle (lights on at 07:00 for all experimental studies). During homecage alcohol consumption studies, mice had *ad libitum* access to food and water. For Pavlovian and instrumental studies, mice were maintained at 85% of their baseline body weight under food restriction. During lickometer studies, mice were mildly water-restricted by removing water bottles ~5 hours prior to testing. Behavioural testing occurred during the light phase.

### Surgeries and viral injections

To monitor dynamic neural activity in Acb, an adeno-associated virus encoding a calcium sensor (AAV9-syn-GCaMP7f) was injected into the Acb, followed by a gradient refractive index (GRIN) lens implanted above the injection site (details in Supplementary Methods).

### Drugs

Alcohol solutions (15%) were prepared by diluting 100% ethanol (Chem-Supply Pty. Ltd; EA043-2.5 L) with tapwater. PF-05231023, a long-acting FGF21 analogue (AdooQ Biosciences, CA, USA), and Exendin-4 (3-39; Bachem; Ex4), a long-acting GLP-1 analogue, were dissolved in saline (0.5% v/v DMSO for PF-05231023 in Experiments 1 and 4 only). PF-05231023 required three cycles of 15-minute warm water baths (36 °C) and sonication to dissolve. Doses were based on previous studies in alcohol-drinking mice and non-human primates [24]. Vehicle was saline (0.5% v/v DMSO in Experiments 1 and 4 only). Drugs were administered intraperitoneally at 10 ml/kg. Injections occurred immediately, 60 minutes or 2-4 hours prior to 24 hr homecage access, cue-approach/lever pressing, and lickometer tests, respectively.

### Cellular-resolution calcium imaging

Calcium imaging of Acb neurons during alcohol consumption was synchronised with lick-event timestamps in mice (N = 8) with correctly placed virus and GRIN lens (Supplementary Figure, S6). Detailed methods are provided in the Supplementary Methods.

### Procedure

Detailed methods are provided in the Supplementary Methods.

#### Experiment 1. Effect of PF-05231023 on homecage alcohol consumption

Male and female mice received 3 weeks of 24-hour intermittent alcohol access (i.e 24-hour access to alcohol on Mondays, Wednesdays, and Fridays). Test occurred between Weeks 4-8. Mice received vehicle, 1 mg/kg, 3 mg/kg or 10 mg/kg PF-05231023 at the start of each week in a within-subjects Latin square design to control for order and time effects. Intake was measured at 24 h, 72 h and 120 h post treatment.

#### Experiment 2. Effect of PF-05231023 on response to alcohol and sucrose cues

Male and female mice were trained to associate auditory cues with alcohol (CS1) or sucrose (CS2). They were trained in two blocks: sucrose cue training (CS+ , CS-; 15 trials/cue/session; 16 sessions, daily) then alcohol cue training (CS+ , CS-; 10 trials/cue/session; 16 sessions, daily). As such mice were trained with two unique CS+ cues conditioned to sucrose and alcohol (counterbalanced) and a third cue that served as a CS- for both rewards. Between the two blocks they received 3 weeks of 12-hour intermittent access to alcohol. They were tested under 10 mg/kg PF-05231023 and vehicle in the following manner: CS1 treatment 1 → 48 hr washout → CS1 treatment 2 → 6 day washout → CS2 treatment 1 → 48 hr washout → CS2 treatment 2, where CS1 and CS2 are sucrose and alcohol cues (counterbalanced order); and treatment 1 and treatment 2 are drug and vehicle (counterbalanced order). Test comprised 20 trials (10 CS + , 10 CS-, pseudorandom order, VI range 100-120 s). Subsequently, mice were retrained with the sucrose CS+ (3 sessions) and alcohol CS+ (3 sessions). They were then tested in the same manner for the effects of the lower dose, 3 mg/kg PF-05231023 (Fig. 2A).

#### Experiment 3. Effect of PF-05231023 on progressive ratio – alcohol and sucrose

Male mice received 6 weeks of intermittent access to alcohol. They were tested on a PR schedule under one of four treatments, counterbalanced in a repeated measures Latin-square design: vehicle, PF-05231023 (3 mg/kg), Exendin-4 (Ex4; 2.4 µg/kg), and combined [PF-05231023 (3 mg/kg) + Ex4(2.4 µg/kg)]. Mice received one PR test per week. They were retrained on random ratio 10 (RR10) at 2 and 4 days post-test to maintain a stable level of lever pressing across testing weeks. They were then tested in a similar manner under one of three treatments: vehicle, PF-05231023 (10 mg/kg), and combined [PF-05231023 (10 mg/kg) + Ex4(2.4 µg/kg)]. The Exendin-alone condition was not included in the second drug factorial design as it was confirmed from the previous round that the 2.4 ug/kg dose had no effect on progressive ratio responding for alcohol in this preparation. In a separate cohort, alcohol-naive male mice were tested on a PR schedule for sucrose reward under PF-05231023 (10 mg/kg) and vehicle (one test per week, maintained on RR10 between tests).

#### Experiment 4. Effect of PF-05231023 on cellular transients in Acb during alcohol consumption

Male mice had 20 min access to alcohol from a lickometer spout in an experimental chamber. Recordings of Acb task-related cellular activity were acquired on Days 5-8. They received saline to acclimatise to the injection procedure on Day 5, then Vehicle and PF-05231023 (10 mg/kg) on Days 6 and 7, respectively.

### Data analyses and statistics

We analysed behavioural and calcium imaging data using custom scripts in R and Python, respectively. Inferential statistics were based on ANOVA using a planned set of orthogonal contrasts for repeated-measures comparisons between treatment groups using PSY statistical program (Bird, 2004), unless otherwise specified. Notably, in Experiment 1, data were analysed separately for each sex, while in Experiment 2, sucrose and alcohol behaviours were examined independently. Specific analyses are described in Supplementary Methods.

## Results

Across experiments mice consumed stable and moderate levels of alcohol during homecage two bottle choice (Supplementary Table 1).

### Experiment 1: PF-052131023 reduces alcohol consumption

**Fig. 1:**
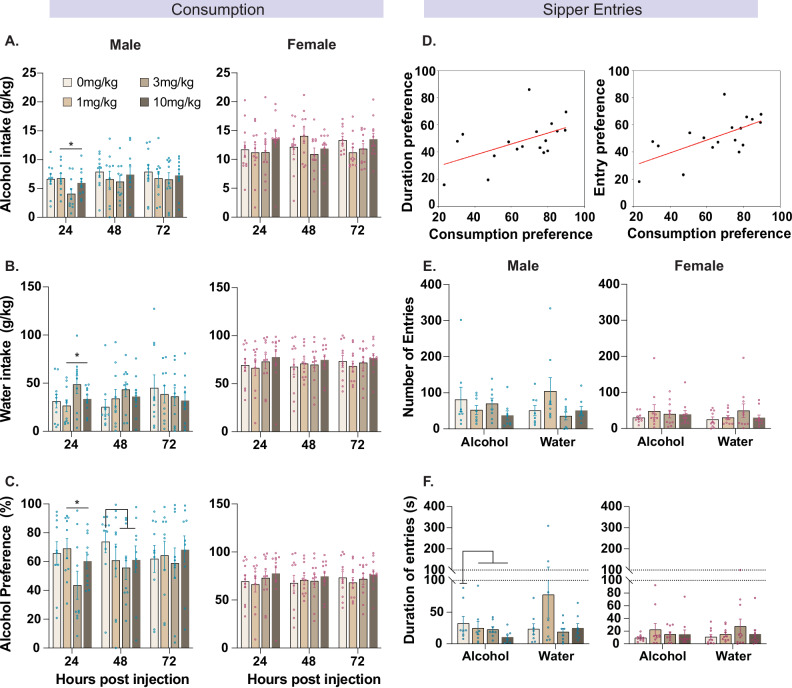
Effects of PF-05231023 (0 mg/kg, 1 mg/kg, 3 mg/kg, 10 mg/kg) on alcohol consumption. **A** Alcohol consumption, **B** Water consumption and **C** Percentage alcohol preference in male and female mice at test. **D** Scatterplot of consumption and entry duration/number preference on the last three days of intermittent access prior to test. **E** Number of entries and **F** duration of entries in male and female mice at test. Data are means ± SEM. *p < 0.05. N = 21 (male n = 10).

We first assessed the dose-response effect of PF-05231023 on alcohol consumption in male and female mice. We excluded two mice (n = 2 males) that exhibited low and unstable alcohol consumption prior to test. There was a dose- and sex-selective effect of PF-05231023 (Fig. 1). In male mice there was a U-shaped dose-response curve when consumption was measured 24 hours after injection. Specifically, alcohol intake and preference was significantly reduced following 3 mg/kg PF-05231023 (Drug quadratic trend: alcohol intake, F (1,9)  = 9.381, p = 0.014; alcohol preference, F (1,9) = 8.955, p = 0.015, Fig. 1A and C). There was no main effect of the drug compared to vehicle at 24 hours post-injection (Drug main effect: alcohol intake, p = 0.228; alcohol preference, p = 0.223), and there were no differences in alcohol intake and preference under 1 mg/kg and 10 mg/kg PF-05231023 (Simple effect: alcohol intake, p = 0.197; alcohol preference, p = 0.216). PF-05231023 also attenuated alcohol preference but not intake 72 hours after treatment, which did not follow a U-shaped curve (Drug main effect: alcohol preference, F(1,9) = 5.749, p = 0.040; alcohol intake, p = 0.217; Drug quadratic trend: alcohol intake, p = 0.428; alcohol preference p = 0.491, Fig. 1C). There was no effect of PF-05231023 on preference or intake at 120 hours after treatment (all contrasts: intake, p > 0.087; preference, p > 0.214). Critically, the effect of treatment was not due to a non-specific decrease in liquid consumption because there was an inverse U-shaped dose-response effect of PF-05231023 on water consumption 24 hours following treatment (Drug quadratic trend: F(1,9) = 6.584, p = 0.030; Drug main effect: p = 0.440; 1 mg/kg or 10 mg/kg simple effects: *p*s > .087; Fig. 1B). This effect did not persist at 72- and 120-hours post-treatment (all contrasts, p > 0.087).

In females, there was no effect of PF-05231023 on measures of alcohol (all contrasts: alcohol intake, p > 0.120, alcohol preference, p > 0.559) or water consumption (p > 0.281). We also did not find any effect of the drug on body weight of animals (all contrasts: females p > 0.300, males p > 0.80). Together these findings suggest that PF-05231023 has a dose-dependent effect on alcohol consumption and preference in male but not female mice.

We next analysed sipper entry data to determine whether PF-05231023 altered homecage drinking behaviours more broadly. Sipper entry tracked consumption of alcohol and water (preference scores) because preference on the last three days of intermittent access prior to test correlated with frequency (R^2^ = 0.40, p = 0.01) and duration of entries (R^2^ = 0.27, p = 0.03, intermittent access days 7-9) (Fig. 1D). We focused analyses on the first day of test (24 hr post injection) given that treatment effects were largest at this timepoint. Our data indicated a dissociation in the effects of PF-05231023 on approach to the sipper and consumption of liquids in male but not female mice. In males under vehicle, sipper entry and duration preference linearly tracked consumption preference (Entry preference, R^2^ = 0.588, p = 0.016; Duration preference, R^2^ = 0.619, p = 0.012) whereas under any tested dose of PF- 05231023 there was no significant linear relationship between consumption preference and approach to the sipper (All Contrasts: Entry preference: R^2^ < 0.385, ps>0.056; Duration preference, R^2^ < 0.217, p > 0.175: Supplementary Table, S2). We did not find this effect in females, although the linear relationship varied across doses of the drug and vehicle (Supplementary Table, S2). This suggests a dissociation between the effects of PF- 05231023 on consumption and sipper approach. Interestingly under drug, male (Main effect: F(1,9) = 7.034, p = 0.026, quadratic trend, p = 0.623) but not female mice (Main effect: p = 0.302, quadratic effect: p = 0.558) spent more time at the alcohol sipper (Fig. 1E). These effects did not extend to other measures because there was no significant effect of the drug on the number of alcohol sipper entries (females: p = 0.637, males: p = 0.115, Fig. 1F) or average entry duration (females: p = 0.310, males: p = 0.935) and no quadratic trend on either measure (Entries: females: p = 0.097, males: p = 0.820; Average duration: females: p = 0.976, males: p = 0.448). We found no effect of PF-05231023 at the water sipper on the number of entries, duration of time, or average entry duration (All contrasts, females: *p*s > 0.102; males: *p*s > 0.215).

### Experiment 2: PF-05231023 reduces alcohol-cue approach without affecting sucrose-cue approach

**Fig. 2:**
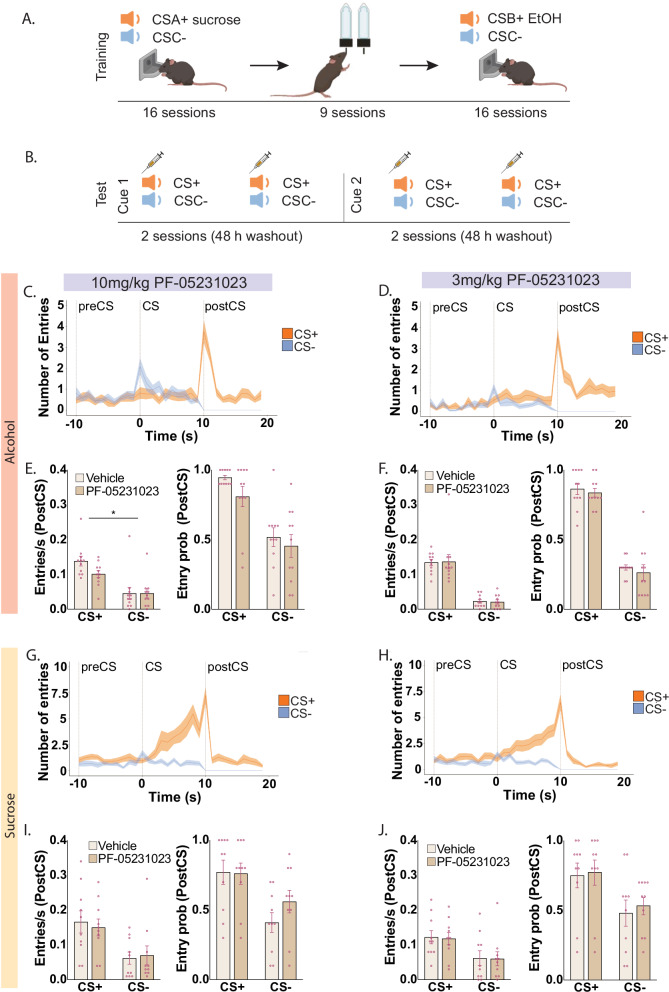
Effects of 3 mg/kg and 10 mg/kg PF-05231023 on approach to alcohol and sucrose paired cues. Note alcohol (N = 11, male: n = 5) and sucrose (N = 10, male: n = 6) responding were analysed separately. **A** Schematic of training and **B** test procedure of conditioned approach to alcohol and sucrose paired cues. Cue 1 and Cue 2 are alcohol and sucrose cues counterbalanced across mice. Treatment order was counterbalanced. **C** Magazine entries during alcohol CS+ and CS- trials separated into minute time bins including the preCS (-10-0 seconds), CS (0-10 seconds) and postCS (10-20 seconds) under 10 mg/kg and **D** 3 mg/kg PF-05231023. **E** Frequency and probability of magazine entry during presentation of alcohol rewards (postCS) during alcohol postCS+ and postCS-under 10 mg/kg PF-50231023 and **F** 3 mg/kg PF-05231023. **G** Magazine entries during sucrose CS+ and CS- trials separated into minute time bins including the preCS (-10-0 seconds), CS (0-10 seconds) and postCS (10-20 seconds) under 10 mg/kg PF-05231023 and **H** 3 mg/kg PF-05231023. **I** Frequency and probability of magazine entry during presentation of sucrose rewards (postCS) under 10 m/kg PF-05231023 and **J** 3 m/kg PF-05231023. Data are means ± SEM. *treatment x cue interaction, p < 0.05.

Next, we assessed the effects of PF-05231023 on conditioned approach to an alcohol-predictive stimulus. We first trained mice with sucrose-cue approach. As expected, mice showed significant conditioned approach during the sucrose cue (CS + > CS-, frequency, F(1,9) = 17.622, p = 0.002; probability, F(1,9) = 137.143, p < 0.001; S3). They then received intermittent homecage access to alcohol followed by training with an alcohol-paired cue (Fig. 2A). However, despite equivalent training, mice did not acquire conditioned approach during the alcohol cue (last three days of training: frequency: p = 0.222; probability: p = 0.717). However, they showed heightened responses to both CS+ and CS- compared to baseline (F(1,10) = 7.22, p = 0.019) and distinguished cues based on alcohol availability at CS+ offset (Fig. 2C, D). For this reason, we applied separate and different analyses to assess the effects of PF-05231023 on responses to sucrose and alcohol cues.

On alcohol cue tests (Fig. 2C–F), approach behaviour was significantly elevated during the CS (Cue main effect: F(1,10) = 15.454, p = 0.003) confirming the learned significance of these cues. Approach responses during alcohol delivery at CS+ offset was specifically elevated relative to control CS- (frequency: F(1,10) = 172.153, p < 0.001; probability, F(1,10) = 230.440, p < 0.001). While there was no effect of PF-05231023 on approach during the CS (10 mg/kg: p = 0.514; 3 mg/kg: p = 0.591), PF-05231023 significantly reduced the frequency of approach during alcohol delivery at the highest dose of 10 mg/kg (treatment x cue, frequency: F(1,10) = 6.817, p = 0.026; probability: p = 0.333, Fig. 2E). There were no effects following the 3 mg/kg dose (frequency: treatment x cue, p = 0.866; probability: treatment x cue, p = 0.905; Fig. 2F).

On sucrose cue tests, mice discriminated sucrose CS+ from control CS- cues, showing elevated approach during the CS+ (frequency: F(1,9) = 5.814, p = 0.039; probability: p = 0.096) and during sucrose delivery at CS+ offset (frequency: F(1,9) = 5.912, p = 0.038; probability: F(1,9) = 8.69, p = 0.016). Critically, PF-05231023 did not affect conditioned approach during the sucrose-predictive cue (10 mg/kg or 3 mg/kg; all *p*s > 0.072) or sucrose delivery at either dose (10 mg/kg or 3 mg/kg; all *p*s > 0.129; Fig. 2G–J).

Together these findings show that PF-05231023 dose-dependently attenuates cue-elicited responses during alcohol availability without affecting similar responses to a sucrose-paired cue. Thus, PF-05231023 may impart selective effects on alcohol-motivated behaviour.

### Experiment 3: PF-05231023 attenuates alcohol seeking under a progressive ratio schedule

**Fig. 3:**
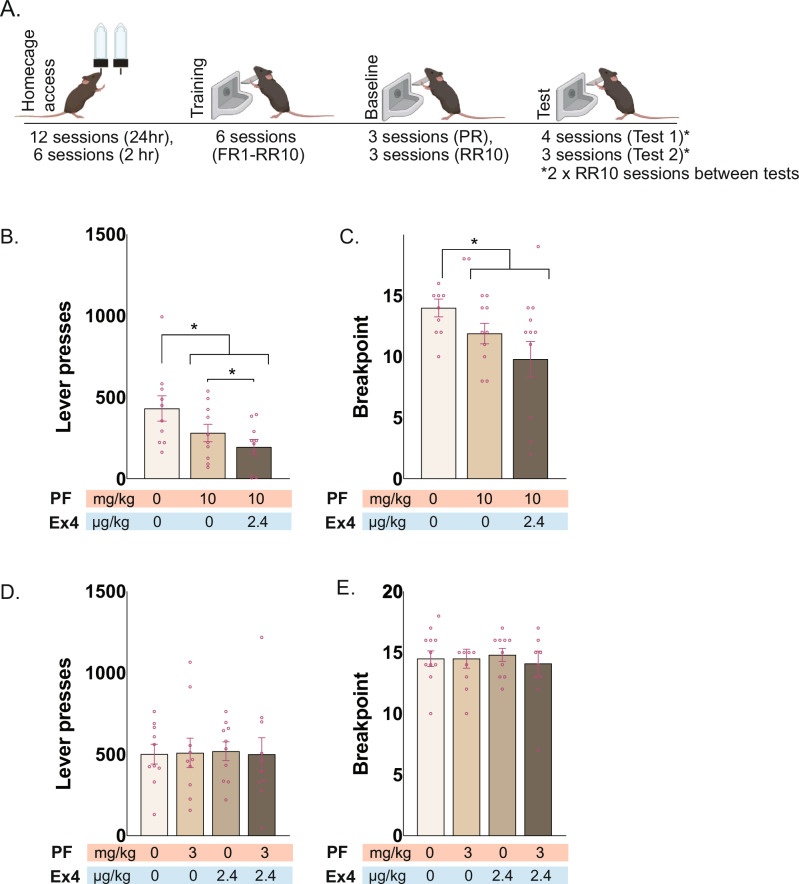
Effects of PF-05231023, Exendin-4, and combination treatment on progressive ratio test of alcohol seeking in male mice (N = 10). **A** Schematic of alcohol access, training and progressive ratio test. Operant schedules: Fixed ratio (FR); Random Ratio (RR); Progressive Ratio (PR). **B** Effects of 10 mg/kg PF-05231023 alone and in combination with 2.4 ug/kg Exendin-4 on total lever pressing and **C** breakpoint. **D** Effects of 3 mg/kg PF-05231023, 2.4 ug/kg Exendin-4, and combination on total lever pressing and **E** breakpoint. Data are means ± SEM. *p < 0.05.

Next, we assessed the effects of PF-05231023 on motivation to respond for alcohol and sucrose using a progressive ratio schedule in male mice (Fig. 3A). Recent approaches using dual FGF21/GLP-1 agonists have shown improved effects on glycemic control, weight-loss and biomarkers of liver function [16, 17] though the mechanisms underlying their complementary actions are not well-described. Nonetheless based on prior preclinical studies implicating both GLP-1 and FGF21 on reducing alcohol consumption [6, 24] we expected this combination to modulate alcohol seeking and to assess whether its effects would be different than the effects of its individual components. We tested PF-05231023 in combination with a low dose of the GLP-1 agonist, Exendin-4, previously shown to minimise off-target effects on locomotor performance [6].

During brief 2-hr homecage access, mice consumed 0.92 ± 0.10 g/kg (mean ± SEM) alcohol solution on average. However, under postprandial conditions (described in supplementary methods), this increased to 2.68 ± 0.51 g/kg / 2 hr (F(1,9) = 18.45, p = 0.002), confirming the efficacy of this state in enhancing alcohol consumption.

During training, lever-pressing for alcohol increased (F(1,9) = 105.041, p < .001). On progressive ratio tests with 10 mg/kg PF-05231023, we found reduced motivation for alcohol, indicated by lower breakpoints (Vehicle vs. Treatment: F(1,9) = 8.840, p = 0.016) and fewer lever presses overall (Vehicle vs. Treatment: F(1,9) = 7.017, p = 0.027). Combined PF-05231023/Exendin-4 did not significantly differ from PF-05231023 alone on breakpoints (F(1,9) = 3.516, p = 0.090) but further reduced overall lever pressing relative to PF-05231023 alone (Fig. 3B, C). Interestingly, 10 mg/kg PF-05231023 had no impact on breakpoint or lever pressing for sucrose rewards suggesting a degree of specificity to alcohol seeking (Supplementary Results, S1, Figure S4).

Notably, PF-05231023’s effects were dose-related. No differences in breakpoint or lever pressing were observed at 3 mg/kg for PF-05231023, or in combination with Exendin-4 (p > 0.60, Fig. 3D, E). Exendin-4 was also ineffective when tested alone (F < 1). Together these findings suggest enhanced efficacy of PF-05231023 when combined with a subthreshold dose of GLP-1 agonist.

PF-05231023 also dose-dependently reduced 1-hour food intake prior to test (10 mg/kg: Vehicle vs. Treatment: F(1,9) = 10.250, p = 0.011; PF-05231023 alone vs combined: p = 0.526; 3 mg/kg: p > 0.31). Importantly, food reduction at 10 mg/kg was unrelated to task performance (Breakpoint: r = −0.478, p = 0.163; Lever presses: r = −0.251, p = 0.484), indicating dissociable effects of PF-05231023 on alcohol motivation and food consumption. Finally, no changes were observed in lever-pressing microstructure (inter-press intervals and post-reward pauses) or latency to first lever press across doses or treatments (p > 0.143 for 10 mg/kg; p > 0.089 for 3 mg/kg; Supplementary Table, S5)

### Experiment 4: Cellular imaging of Acb and microstructure of alcohol consumption

**Fig. 4:**
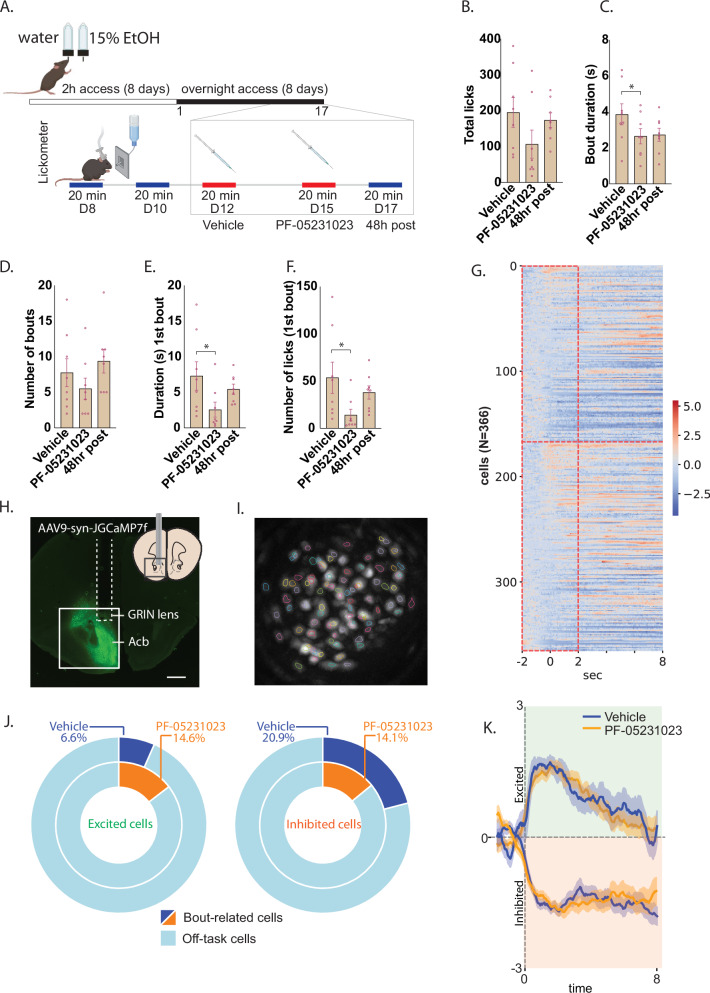
PF-05231023 disrupts lick microstructure during alcohol consumption and modulates consumption-related activity in Acb neurons in male mice (N = 8). **A** Schematic of training and test procedure. **B** Effects of 10 mg/kg PF-05231023 on total number of licks, **C** bout duration and **D** number of bouts across the session; **E** Duration and **F** Number of licks on the 1^st^ bout. **G** Heat map of mean z-scored GCaMP7f peri-bout activity for all neurons during Vehicle (cells 1-199) and PF- 05231023 (cells 200-366). **H** Example virus expression and GRIN lens position in Acb. **I** Example maximum ΔF/F image and identified neurons. **J** Fraction and **K** Z-score GCaMP7f activity of excited and inhibited neurons. GCaMP7f data are normalised to baseline. Data are means ± SEM. *p < 0.05.

Male mice received the following intermittent homecage alcohol access schedule: 2 hr access for 8 days (starting 10:00), 24 hr access for 4 days, then 16 hr (starting 17:30) access for all subsequent (testing) days. On test days mice were allowed 20 minutes to freely consume alcohol in a lickometer chamber (session starting at ~10:00). We examined how 10 mg/kg PF-05231023 affects alcohol consumption by imaging calcium dynamics in Acb neurons during voluntary alcohol intake (20 min sessions; Fig. 4A). We assessed lick patterns and corresponding activity of pooled Acb neurons, given that Acb is a critical target through which KLB + BLA-projecting cells modulate alcohol consumption [24].

Mean total licks (±SEM) were 195.1 ± 41.3 (Vehicle), 106.8 ± 39.3 (PF-05231023), and 173.6 ± 20.1 (48-hour post-test, no treatment). While PF-05231023 did not significantly reduce total licks (F(1,7) < 1), it disrupted the microstructure of drinking, reducing lick bout duration (Vehicle vs. PF- 05231023: F(1,7) = 8.17, p = 0.024; PF-05231023 vs. 48-hour post: p = 0.083) without significantly affecting bout frequency (p > 0.05; Fig. 4B–D). Effects were more pronounced for the first bout: PF-05231023 reduced both lick count and bout duration (Lick count Vehicle vs. PF-05231023: F(1,7) = 7.156, p = 0.032; Lick count PF-05231023 vs. 48-hr post: F(1,7) = 22.466, p = 0.002; Bout duration Vehicle vs. PF-05231023: F(1,7) = 6.648, p = 0.037; Bout duration PF-05231023 vs. 48-hr post: F(1,7) = 10.974, p = 0.013). PF-05231023 did not alter movement frequency (F(1,7) < 1) or subsequent homecage alcohol consumption (F(1,6) < 1). The selective effect of PF-05231023 on bout size is consistent with PF-05231023 possibly targeting pre-ingestive evaluative processes (e.g. palatability) [31].

Correspondingly, many Acb neurons exhibited time-locked inhibition at lick bout onset. Under Vehicle, 25.1% (42/167 cells) showed inhibited GCaMP7f transients, while 20.6% (41/199 cells) did so with PF-05231023. Conversely, fewer neurons were excited (Vehicle: 12.0%, 20/167 cells; PF- 05231023: 15.1%, 30/199 cells). While there were more inhibited than excited cells under Vehicle (two-proportion Z test, p = 0.002), PF-05231023 abolished this imbalance (p = 0.15). Analyses of the first lick bout revealed similar trends, with more cells exhibiting inhibition under Vehicle (21%, 35 /167 cells inhibited; 7%, 11/167 cells excited) (p < 0.001) but not under PF-05231023 (14%, 28/199 cells inhibited; 15%, 29/199 cells excited; p = 0.77, Fig. 4J, K). Critically, PF-05231023 did not alter the temporal profiles of bout-related transients (Fig. 4K) suggesting that a primary effect of PF-05231023 is to diminish the relative contribution of inhibited cells.

**Fig. 5:**
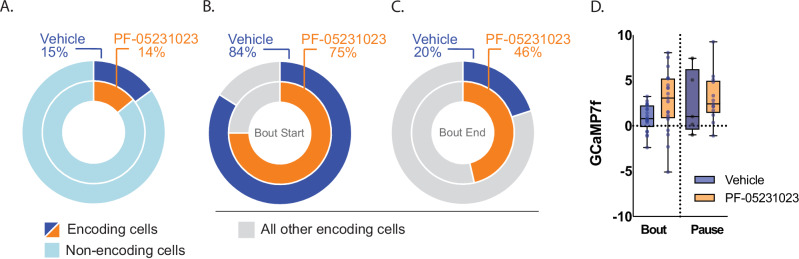
PF-05231023 differentially recruits cells around alcohol consumption in male mice (N = 8). **A** Fraction of neurons significantly modulated around lick bouts. **B** Fraction of bout-modulated cells recruited during bout onset (bout) and **C** bout offset (pause). **D** Mean GCaMP7f kernel activity over a 2 s window following bout onset and offset. *p < 0.05.

We next assessed cells classified on the basis of their activity around the initiation and termination of a lick bout (ie, bout vs. pause cells). To disentangle bout onset and offset signals, we used an encoding model that removed the linear component of closely-timed events from the target event [32]. We found similar proportions of significantly modulated cells between Vehicle (15.0%, 25/167) and PF-05231023 (14.1%, 28/199; Fig. 5A). When bout- and pause-encoding cells were separately examined, the percentage of bout cells was also similar across treatments (expressed as a proportion of significantly modulated cells, Vehicle: 84%; PF-05231023: 75%, p > 0.05, Fig. 5B). However, mean kernel GCaMP7f activity of these bout cells was significantly higher following PF-05231023 than Vehicle (F(1,40) = 5.251, p = 0.027; Fig. 5C). PF-05231023 also increased the proportion of pause cells (Vehicle: 20%; PF-05231023: 46.4%, p = 0.04) without affecting their kernel activity (F < 1; Fig. 5B, C). Finally, we note that bout- and pause-cell overlap was minimal under Vehicle (4%) but increased with PF-05231023 (21.4%, p = 0.06).

## Discussion

FGF21 has been shown to regulate alcohol consumption across species [23–27]. Here, we demonstrate its broader influence on alcohol-motivated behaviour and identify potential mechanisms for this regulation, including reducing alcohol palatability and modulating consumption-related Acb neuron activity.

### PF-05231023 and alcohol intake

Our findings confirm that PF-05231023 reduces alcohol intake, consistent with prior studies using the same dose in a similar intermittent access model [24]. However, unlike previous reports, we did not observe persistent treatment effects, though we note differences in dosing schedules and timing. We used a single 3 mg/kg dose following 3 weeks of intermittent access where others have used bi-weekly treatments of the same dose from the initiation of access [24]. Notably, we found PF-05231023 reduced alcohol intake only in male mice, contrasting earlier findings of indiscriminate effects of PF-05231023 on alcohol intake across sexes [24].

The cause of this sex difference remains unclear but may relate to reported sex-specific metabolic effects of exogenous FGF21 [14, 33–36], possibly linked to differences in FGF21 receptor expression or liver status following dietary changes [14, 35]. In our study, mice had stable and moderate levels of drinking before treatment. Whether similar sex distinctions occur in mice with chronic access to alcohol is not well-characterised but is an important consideration given the hedonic [37] and neurobiological changes associated with prolonged alcohol exposure and the higher alcohol consumption in female mice relative to males.

### FGF21 and preparatory appetitive behaviours

To date, studies have focused on FGF21’s role on consumption but alcohol-associated cues have substantial effects on craving and alcohol-motivated actions. For example, in humans, alcohol cues increase subjective craving, impair inhibitory control, bias attentional processes and increase consumption [38–41]. Similarly in rodents, alcohol cues can invigorate instrumental alcohol seeking and drive relapse like behaviour [42–44]. The effects of FGF21 on reward-predictive cues have not been previously shown. Here, we show that PF-05231023 had no effect on approach maintained by sucrose cues. This is despite the role for FGF21 in regulating sweet preference [23], and suggests a dissociation in the actions of FGF21 under opportunities of voluntary *ad lib* consumption and cue-controlled approach. Critically, PF-05231023 impaired alcohol approach without reducing the number of trials that elicited approach. This suggests that under these conditions, the primary effect of PF-05231023 may involve reducing the behavioural vigor associated with alcohol anticipation. However, because elevations in approach for alcohol were well-aligned to its availability, further studies are needed to discern the effects of exogenous FGF21 on the incentive and signalling properties of alcohol-related cues.

While the effects of PF-05231023 on alcohol approach are closely tied to the moment of consumption, PF-05231023 also reduced the willingness to work for alcohol but not sucrose on a progressively increasing schedule of reinforcement in male mice, a finding that clearly demonstrates the reward-selective effects of PF-05231023 in a manner temporally dissociated from consumption. Importantly, PF-05231023 did not alter latency to first lever press, suggesting that reductions in responding in alcohol tasks were not due to general impairments related to task engagement or motor initiation. Given that alcohol approach effects were found across sexes, possible sex-related dissociations in PF-05231023 effects on seeking versus taking is warranted in future studies. Moreover, in male mice, PF-05231023 disrupted the microstructure of alcohol consumption, specifically reducing the size but not number of lick bouts - a dissociation which has been associated with a reduction in pre-ingestive evaluative processes and reward palatability [31]. As such, a change in hedonic responses to alcohol may be one mechanism through which PF-05231023 reduces alcohol intake.

Notably, there was a complex dose-response relationship across our studies. We found that the highest dose was effective on cue-approach, lever-pressing, and lickometer tests, yet ineffective for ad lib 24 hr homecage consumption, which required a lower dose. This may be due to possible differences in endogenous FGF21 levels at test associated with differences in actual levels of intake between tasks, given that alcohol strongly stimulates hepatic FGF21 release [20, 45, 46]; or associated with the food-restricted status of mice undergoing behavioural tests, given that food restriction can elevate FGF21 hepatic expression and plasma levels [47] and that diet induced-elevations in FGF21 can lead to an FGF21 resistant-like state [48]. How dose-response functions shift under varying testing conditions warrants further investigation but together, our findings highlight the importance of testing multiple doses when working with FGF21 across different tasks.

### Combined targeting of FGF21 and GLP-1 reduces alcohol motivation

The broad effects of PF-05231023 across multiple forms of alcohol-motivated behaviour parallel those of gut-brain signalling hormones, like GLP-1, which can reduce self-administration [6, 49, 50], conditioned place preference [6], and relapse [50–52]. Critically, our findings on combining GLP-1 and FGF21 are encouraging for subsequent studies to pursue this work in the form of multiple-dose assays to formally assess synergistic dose interactions. Treatment with a sub-threshold dose of the GLP-1 agonist, Exendin-4, augmented the effects of PF-05231023 on reducing total lever presses on a progressive ratio test–a sensitive indicator of motivation over the canonical breakpoint when using an exponential response requirement [53]. We note that in contrast to previous studies, Exendin-4, when tested alone, had no effect on instrumental performance [6, 50] which may be due to fasting-dependent changes in GLP-1 receptor localisation or signaling [54].

Together these findings concur with evidence of complementary and inter-dependent mechanisms of action between FGF21 and GLP-1 [55–58], and recent developments of GLP-1, FGF21 dual agonist compounds [16, 17] in the context of weight loss and insulin sensitivity. Our findings suggest that combination peptide agonist approaches may similarly be of benefit in the treatment of AUD.

### FGF21 reduces consumption-related pauses in Acb cells

A wealth of literature implicates overlapping neurobiological substrates mediating FGF21 effects on alcohol consumption and those mediating appetitive behaviour. For example, Pavlovian conditioned approach depends, in part, on glutamatergic projections from basolateral amygdala (BLA) to the Acb [28, 59–62]. Optogenetic excitation of this BLA→Acb pathway can arrest both alcohol consumption and approach to an alcohol cue [28]. Crucially, PF-05231023 increases excitability of KLB-expressing BLA→Acb neurons, and FGF21 suppresses alcohol, but not sucrose, consumption via these BLA KLB+ neurons [24]. Our findings further highlights this overlapping role for the Acb. Here we report that distinct subsets of Acb cells are time-locked to lick bouts; that for a majority of these cells, activity is inhibited and that the recruitment of these inhibited cells is undermined by treatment with PF- 05231023. Moreover, under PF-05231023, more cells were modulated by the onset of pauses in drinking, consistent with the reduction in bout size on test. Finally, while PF-05231023 did not significantly alter the number of cells recruited during the onset of a lick bout, it increased the activity of these cells. While it is difficult to relate activity to behaviour on a bout-by-bout level due to variations in the number of bouts across mice, these findings fit within an established role for Acb in the control over consumption and learned appetitive behaviour [29, 30, 63–65]. Moreover, they suggest that a retuning of specific subsets of Acb cells associated with initiating and terminating consumption may be a key mechanism through which PF-05231023 exerts control over alcohol intake; a theory that invites further scrutiny and future imaging studies examining the interactions of FGF21 and GLP-1 mimetics.

FGF21 is one of two endocrine FGFs (FGF21 and FGF19) that signal via an FGF receptor and KLB receptor complex. These FGFs share similar functions associated with the regulation of weight, glucose and insulin levels [66]. While the role of FGF19 on alcohol is yet to be described, FGF21, unlike FGF19, has non-mitogenic properties, making it a more attractive candidate for therapeutic development [67].

## Conclusions

PF-05231023 reduces alcohol consumption, approach, and motivation, likely through reductions in hedonic responses to alcohol and via modulation of Acb neuron activity to control intake. These findings highlight FGF21’s potential as a therapeutic target for AUD.

## Supplementary information

Supplementary Material

## Data Availability

Data will be shared and made available upon reasonable request. Code and further information related to the event-encoding model will be shared upon reasonable request and made publicly available on GitHub as of the date of publication.
